# Producing Child-Centered Interventions: Social Network Factors Related to the Quality of Professional Development for Teachers of Autistic Students

**DOI:** 10.3390/socsci10120453

**Published:** 2021-11-25

**Authors:** Elizabeth McGhee Hassrick, Jessica Suhrheinrich, Patricia Schetter, Allison Nahmias, Melina Melgarejo, Jennica Li, Jonas Ventimiglia, Yue Yu, Aubyn Stahmer

**Affiliations:** 1 A.J. Drexel Autism Institute, Drexel University, Philadelphia, PA 19104, USA;; 2 Department of Special Education, San Diego State University, San Diego, CA 92182, USA;; 3 Departments of Psychiatry, Davis MIND Institute, Psychology & Human Development, University of California, Sacramento, CA 95817, USA;; 4 Stony Brook Medicine, Stony Brook, NY 11794, USA;

**Keywords:** Autism Spectrum Disorder, teacher training, professional development, education, social networks, child-centered evidence-based practice, implementation science

## Abstract

Autistic students benefit from child-centered goals that align with evidence-based practices (EBPs) that meet their individualized needs, however, most teachers are not trained in how to implement autism-specific EBPs. The challenges do not lie with teachers alone. Professional development (PD) providers, such as district or regional autism experts who train and coach teachers on how to implement autism-specific EBPs, face barriers accessing the needed supports to conduct high-quality PD and lack experience with individualizing their methods for training and coaching teachers. When PD providers have networks of professional support, they can potentially gain access to resources to provide successful individualized coaching for teachers. No research has measured the impact of the social networks of PD providers on their performance as coaches in classrooms for teachers of autistic students. To test the hypothesis that social network resources can impact the performance of PD providers who coach teachers how to use EBPs for their autistic students, we conducted social network analysis with PD providers. Findings suggest that network factors were associated with the self-reported performance for PD providers. PD providers who have more people in their networks who were autism EBP experts, as well as more people in their networks who supported them with how to individualize their PD efforts to specific teachers or districts, had higher performance as teacher coaches. We discuss future research about how to support network development for PD providers and policy implications.

## 1. Introduction

The pressing need to provide instructional supports for autistic students is considerable due to their rise in prevalence (Maenner et al. 2020). Autistic students have highly variable and often co-occurring complex needs that can include seizure management and/or mental health support for anxiety and/or depression (Levy et al. 2010). Cognitive and communication skills also vary greatly, with 30% presenting as minimally verbal (Tager-Flusberg and Kasari 2013) and 30% presenting with intellectual disability (Polyak et al. 2015). Autism must be considered as having different levels of severity along a ‘spectrum’. As a result, autistic students benefit from access to research-supported, evidence-based interventions (EBPs). EBPs are interventions that have been validated through rigorous empirical testing to improve outcomes for children on the spectrum. A systematic review of the scientific literature indicates several specific EBP for autism (e.g., Steinbrenner et al. 2020), however, most teachers are not trained in how to use or select EBPs for their autistic students.

To address this gap in training, teachers can be supported by professional development (PD) providers who could coach them in how to select and implement autism-specific EBPs in their classroom. The evidence supporting coaching for teachers has become more widespread in general education (Kraft et al. 2018), however, individualized instructional guidance for teachers of children with special needs such as autism is lagging behind (Rosenzweig 2009). PD providers face considerable barriers in accessing classrooms and working directly to coach teachers of autistic students. In the United States (US) context, not only are district, regional and state systems often siloed and fragmented in their support for the implementation of evidence-based interventions for special needs students (Fixsen et al. 2012), but PD providers generally have little training about how to conduct high-quality professional development, tailor their training and coaching for specific contexts or access the resources needed to perform PD tasks such as securing access to teacher release time or financial supports for EBP materials.

Social network resources, such as advice about how to tailor PD approaches, navigate schools and secure financial support, could assist PD providers with overcoming many barriers, including how to access the needed resources to most effectively coach teachers in their classrooms about using evidence-based practices (EBPs). To date, social network studies in the US have focused primarily on how networks shape regular education teachers or principals (Coburn 2001; Coburn and Russell 2008; Finnigan and Daly 2010; Frank et al. 2014; Penuel et al. 2012; Spillane 2005), or how connections between school leaders and district contexts impact school improvement (Daly and Finnigan 2012, 2013; Finnigan and Daly 2010). No studies have examined how the social networks of US district, regional or state-level PD providers might impact their performance as coaches in classrooms for teachers of autistic students.

This study asks the research question, “Are the professional social networks of PD providers associated with improved performance in coaching teachers to implement EBPs for autistic students?” We hypothesize that EBP knowledge, coaching support and financial resources embedded in PD provider professional social networks could be associated with higher quality coaching by PD providers.

### 1.1. EBP Intervention in US Public Schools for Autistic Children

Current estimates indicate that 1 in 54 children in the United States have autism (Maenner et al. 2020). As the population of autistic children in public schools has increased, so has the need to provide effective services and supports. In the US, most autistic students receive their interventions and related supports in public school settings, especially students from lower-income families who cannot afford private clinical intervention in community settings (Brookman-Frazee et al. 2010; Wood et al. 2015). Research has identified effective EBP for students with autism (e.g., Steinbrenner et al. 2020), but unfortunately, the integration of EBPs into US public schools remains limited. Variation in EBP implementation for autistic children also exists internationally, with some countries leading the way and others lagging behind (Dillenburger et al. 2014).

Clear facilitators for school-based services for autism, such as EBP training at the intervention level (Sam et al. 2020) and US federal policy mandates to support use of evidence-based interventions for students with disabilities (IDEA, ESSA) at the systems level, have been found to impact EBP implementation (Moullin et al. 2019). However, less understood are how to address implementation challenges in the classroom for autistic students. Teachers not only need to learn EBPs, they also must determine which EBP or combination of EBPs are needed for each autistic student. Coaching from EBP experts familiar with the challenges that teachers face in their classrooms implementing EBPs could help them address the variation in instructional needs for their autistic students and help them individualize their instructional efforts. However, EBP experts for autistic students are often situated at the district or regional level, rather than in schools and classrooms. Implementation support for EBP use with autistic students remains out of reach for most teachers.

### 1.2. Reconfiguring US Educational Systems to Improve EBP Implementation

In the United States Public Education System, it is common for each state to interpret the federal educational regulations and develop additional regulations of their own. Among these are the provisions of special education services for students with identified needs. State and Federal regulations and funding are then allocated to school districts, many of whom are large and can operate their own special education services and supports. The smaller districts often form a consortium for the purposes of delivering special education services and resources. These consortia are known by different names, depending on the State (e.g., Regional Service Areas (RSA), Special Education Local Plan Areas (SELPA), Board of Cooperative Educational Services (BOCES)). Special education consists of many services and supports that are designed to meet a student’s individual and unique needs. They may include things such as speech and language therapy, occupational therapy or specialized academic instruction that can take place within a general education classroom or in a specialized classroom. In the US context, teachers are legally required to identify and select EBPs to help meet the identified needs of their students, as stated in the Individuals with Disabilities Education Act (IDEA 2004). Very limited training is offered through pre-service teacher preparation programs on the EBPs for autism (Morrier et al. 2011), therefore, educators must often rely on in-service professional development to learn about these practices. As a component of special education support, professional development is often provided to the educators within a district by PD providers. These PD providers are commonly professionals who have been classroom teachers, behavior specialists, school psychologists or speech therapists who have specific technical knowledge and skills in a given area or with a specific population, such as students with autism. They are promoted into positions where they are asked to provide training and coaching (PD) to other teachers or educators who need additional skills in working with a specific population of students. Although they may have demonstrated skill and expertise in working with this population, often PD providers are not given any specific training or support related to how to conduct high-quality PD or how to access the resources needed to conduct PD within the educational system.

At the systems level, prior social network research has identified the influence of connections between district leaders and school principals on compliance with district, state or federal mandates. Trusting network connections between school principals and district leaders were predictive of reciprocal best practice relationships (Daly and Finnigan 2012; Finnigan and Daly 2010). In addition, the diffusion of research evidence among districts was found to impact school performance, where under-performing schools were situated in the periphery of information sharing networks due to lower levels of engagement by super-intends in information brokerage (Daly and Finnigan 2013). Attempts to diffuse research-based policy and practices at the district level through program coaching for district-level staff was found to be partially successful, where a few most knowledgeable coaches emerged as central diffusers of research evidence, with most interactions only occurring in formal meetings (Rodway 2019), hampering sustainability. Similarly, state-level entities are broadly understood as providing policy guidance and are not generally identified as intervention providers to teachers in classrooms (Spillane 1998). Studies that investigate the impact of district, regional or state professional development providers on teacher practices in the classroom, usually in the form of external professional development sessions away from the school context, report weak effects (Gamoran et al. 2000). More proximal influences, such as principal leadership, technical resources, professional community and program coherence at the school level, drive instructional quality (Newmann et al. 2000).

Efforts to leverage the proximal influences, or implementation factors, related to effective integration of EBP are relatively new. The field of implementation science developed in response to a need to address research to practice gaps and has been described as the scientific study of methods to promote the systematic uptake of research findings and other evidence-based practices into routine practice, and, hence, to improve the quality and effectiveness of health services (Eccles and Mittman 2006). Systems level interventions are relatively new and are largely part of implementation science efforts to improve evidenced based practices across multiple institutional settings. One example of such an effort in the state of California is CAPTAIN (California Autism Professional Training and Information Network; Suhrheinrich et al. 2020), a statewide interagency collaboration with the goal of scaling up use of EBPs for autistic individuals through targeted implementation supports and coaching. CAPTAIN currently has members representing regionalized special education agencies who serve as PD providers in their regions. CAPTAIN PD providers are required to provide EBP training and coaching to teachers and other educators each year, (a) at least 1 awareness training about autism and EBPs, (b) at least 3 trainings on specific EBPs, and (c) EBP-specific implementation coaching to at least 3 providers or programs. California currently has 1028 school districts divided between 52 counties. For the purposes of Special Education delivery, these school districts are aggregated into 134 Special Education Local Plan Areas (SELPAs), which are regional consortiums responsible for the provisions of special education services and compliance with special education laws. Other US states have similarly organized education systems where special education staff, teachers and students from different schools and districts receive intervention support from regional entities, for example, the Intermediate Unites (called IUs) in Pennsylvania or the Boards of Cooperative Educational Services (called BOCES) in New York State. CAPTAIN has representation from 93% of these SELPAs across the state. Several other states in the USA have similar autism EBP networks which mirror the professional learning community model used in many areas of education.

### 1.3. Using Social Network Methods to Measure Relationships

Social network research has been embraced by multiple disciplines, including education (Bryk et al. 2013) and public health (Valente 2012), to explain collective and individual behavior (Borgatti et al. 2009). Social network analysis uses graph theory, a rigorous, mathematical measurement of patterns of interactions that can be used to identify the relationships and resources that make up the social structures around a person (Wasserman and Faust 1994). In social network theory, the relationships that PD providers have can act as “pipes”, or conduits, through which professional resources flow. Resources can be material, such as financial support to train teachers how to implement EBPs, or less tangible resources, such as expertise about how to work with school leaders to gain access to teachers for coaching and training or EBP expertise about how to help teachers solve problems in their classrooms as they attempt to use new strategies. Mapping the pattern of relationships around PD providers can identify opportunities and constraints related to the social supports and resources that PD providers access from their networks (Wasserman and Faust 1994).

This study investigates the association between the social networks of PD providers who participated in a California state-level initiative (CAPTAIN) designed to increase special education teachers’ implementation of EBP for students with autism and their performance as PD providers. For this study, self-report by PD providers was used to measure performance. The use of PD provider self-report has important limitations, as people’s perceptions of their performance does not capture the impact of their performance on educators and their students with autism. However, a positive relationship has been found between self-assessment and external evaluations in various fields, suggesting the potential benefit of self-report (Biernat et al. 2003; Pisklakov et al. 2014). We were not able to measure PD provider performance as rated by teachers receiving coaching due to funding constraints.

## 2. Methods

PD provider performance, the primary outcome, was measured using participant report of training and coaching requirements completion and quality training and coaching practices. A network survey was used to measure PD providers’ professional networks. We used General Linear Models (GLM) to test the association between the professional support networks of PD providers and their performance. Participant demographics included years in position, EBP knowledge and EBP financial and leadership authority. Each CAPTAIN PD provider was employed by regional consortiums called SELPAs that manage special education services and compliance of districts and schools. SELPAs varied by type, with some SELPAs managing multiple districts and others managing one district. SELPAs also varied in size. These differences were used as control variables in the analysis. See section below for study details.

### 2.1. Study Participants

Study participants were PD providers who participated as CAPTAIN PD providers as part of a SELPA. CAPTAIN PD providers received an annual survey via Qualtrics in October 2017. Prior to starting the survey respondents reviewed a consent form approved by the UC Davis IRB and could consent to having their survey responses used for research purposes. Three hundred and seventeen (96.6%) of the SELPA PD providers consented. Of those, 247 (77.9%) were returning PD providers who completed questions about their success in meeting CAPTAIN training and coaching goals in the previous academic year. A total of 228 returning CAPTAIN PD providers (92% of eligible participants) completed the network survey, however, 2 PD providers represented charter schools, and their data were dropped due to lack of data for the charter school context. Data are presented from 226 returning PD providers from 102 SELPAs. As seen in Table 1, PD providers had a mean of 6 years in their professional role and reported high knowledge of EBPs for autism but less authority for implementing EBPs.

### 2.2. Outcome Variable

CAPTAIN PD providers’ performance was the primary outcome variable. CAPTAIN PD providers self-report about their completion of training and coaching requirements as part of the annual survey. A performance score was calculated for each PD providers member to incorporate both how well they fulfilled their coaching and training requirements (quantity of training and coaching sessions) as well as how often they used quality training and coaching practices.

Training and coaching requirements’ completion (quantity of training and coaching sessions) was measured by asking PD providers to rate whether they fulfilled each of the following three training requirements: (a) the awareness training requirement (at least 1 training per year) was rated on a scale of (0) “I did not conduct any trainings” to (2) “I conducted more than one training”; (b) the EBP training requirement (at least 3 per year) was rated on a scale of (0) “I did not conduct any trainings” to (4) “I conducted more than three trainings”; and (c) the coaching requirement (coach at least 3 teachers or programs per year) was rated on a scale of (0) “I did not provide coaching to any teachers or programs” to (4) “I provided coaching to more than three teachers or programs”.

Quality training and coaching practice use was measured by asking PD providers how often they used the practices, adapted from Joyce and Showers (2002) recommendations for effective professional development, on a scale of (1) “Never” to (3) “Always.” Two quality training questions asked how often the PD providers (1) used videos, showed live demonstrations or role played and practiced the EBP or skill they were training on and (2) tested for content knowledge to see if recipients of training acquired desired information. Two quality coaching questions asked how often the PD providers (1) collected and evaluated data on the student in order to ensure the EBP was having a desired effect and (2) used implementation/fidelity checklists to determine if EBPs were being implemented with fidelity.

Outcome Score. Training and coaching quality outcomes were determined using a formula that multiplied training and coaching requirement (quantity) by training and coaching quality practices and weighted the importance of the training and coaching methods. We weighted training and coaching according to Joyce and Showers (2002) research on teacher use and acquisition of knowledge and skill through professional development. Awareness training was weighted less since it solely provides an overview of EBPs, while EBP training involves training on how to implement a specific EBP. Coaching was weighted more as research indicates it is contributing to the transfer of training to the classroom (Joyce and Showers 2002).

((Awareness training × Use videos, show live demonstrations, or role play and practice) × (0.5)) + ((Awareness training × Test for content knowledge) × (0.5)) + ((EBP Training × Use videos, show live demonstrations, or role play and practice) × (1)) + ((EBP Training × Test for content knowledge) × (1)) + ((Coaching × Use implementation checklists) × (2)) + ((Coaching × Collect and evaluate data) × (2)). Performance scores ranged from 0.00–78.00, with a mean of 41.89 (19.01).

While observational reports from teachers, student or families of PD provider performance or video recorded sessions would add value to the analysis as outcome variables, the study did not have the financial resources required to annually collect such data. Although we were not able to use external observations, studies in various fields have suggested a positive relationship between self-report and external assessment. For example, Desimone and colleagues (Desimone et al. 2010) have suggested, based on their meta-analysis, that teachers’ self-reports on their teaching quality are strongly correlated with classroom observations and teachers’ records. A study that examined physicians’ competence suggested a positive relationship between self-assessment and external assessment in certain competence areas (Biernat et al. 2003). Additionally, studies have suggested that self-report performance can be beneficial through enhancing self-efficacy and self-motivation after self-assessment in other fields (e.g., medical student and resident; Pisklakov et al. 2014).

### 2.3. Egocentric Network Analysis

Egocentric network analysis (Hanneman and Riddle 2005; McCarty 2002; Neal et al. 2015; Wasserman and Faust 1994) is a well-established approach routinely used in publications over the past 40 years for several major social science surveys, including the NSF-funded General Social Survey (1985–2014) (Burt 1984), the National Longitudinal Study of Adolescent to Adult Health (1994–2008) and the National Social Life, Health and Aging Project (2005–2015). Egocentric network measurement includes the number and type of social connections a person has with others (Berkman et al. 2000; Smith and Christakis 2008; Wasserman and Faust 1994). Egocentric networks include the respondent (“ego”) and key people with whom the respondent identifies (“alters”) (Marsden 1990). A “name generator” question (Burt 1984) is asked, and for each name generated, several additional questions are asked that characterize the alters in each participant’s network. For this study, PD providers were asked to identify up to four people who provided coaching and support for their job as a CAPTAIN PD provider. Examples of coaching activities included people who helped the PD provider figure out how to help a struggling teacher, how to determine which EBPs are key for certain students and people who provided the PD provider with good EBP training approaches. PD providers were also asked to identify up to four people who provided financial support for their job as a CAPTAIN PD provider.

Examples included people who helped the PD providers secure dedicated time to provide coaching/mentorship, people who provided funding to buy materials needed for training and implementation (e.g., laptops, projectors, and laminators) and people who were willing to allocate dedicated time and resources (e.g., color printers, laminators) for the teachers the PD providers were coaching/mentoring to support their EBP implementation with their autistic students. This relationship represents a professional tie between the ego and the identified alters. For each of the people named, additional questions focused on the type of support provided (e.g., different types of coaching or financial support) and the level of EBP expertise of each alter, rated using the following scale: No expertise, basic knowledge, some knowledge, average knowledge, above average knowledge and high level of expertise. The PD provider also identified connections among alters. For each unique pair of alters, the PD provider was asked by the network generator, “How close is (Person A) with (Person B)”, using a scale with the following choice selections: Very close; Friendly, but not close; See/talk with each other, but not friendly; not close, Do not know each other; and I do not wish to answer this question. The name generator was limited to four people from each category (coaching, finance) to reduce burden on the participant (max eight), as each additional person named generates additional attribute and connection questions.

Two measures were used to characterize network structure, including network size and high communication density. Network size was measured by the overall number of alters named by the PD providers (max 8). High communication density was measured by dividing the number of actual ties for frequent communication by the number of possible ties. Possible ties refer to all ties that could exist among all identified alters. Four network measures were used to characterize the resources provided by network alters (referred to as network resources), including the number of EBP experts, number of coaching providers, number of finance providers and number of CAPTAIN PD providers and the number of alters listed for each sub network (e.g., coaching network and financial network). To calculate the number of EBP experts, the total number of alters rated as having the highest level of expertise were summed (max 8). To calculate the number of coaching providers, the total number of alters named by the PD providers as coaches were summed (max 4). To calculate the number of financial providers, the total number of alters, up to 4, named by the PD providers as financial supporters were summed. To calculate the number of CAPTAIN people, the total number of alters named by the PD provider who participated in the CAPTAIN intervention as PD providers (past or present) or leaders were summed, with a total of 8 being the max.

### 2.4. US Context Measure

Context characteristics of the SELPAs CAPTAIN PD providers represented were determined based on publicly available data. SELPA type was coded as single-district (serves one school district) or multi-district (serves multiple school districts). Sixty-seven percent of participating PD providers were situated in multi-district SELPAs.

SELPA size was determined by the number of students in each SELPA. Three cut points were created to generate three equivalent groups to code SELPAs as small, medium or large. Small SELPAs had fewer than 25,000 students, medium SELPAs had 25,000–45,000 students and large SELPAs had more than 45,000 students. Moreover, 48 percent of PD providers represented large SELPAs, 31% represented medium SELPAs and 21% represented small SELPAs.

### 2.5. PD Provider Characteristics

PD provider characteristics were collected as part of the annual CAPTAIN survey. PD providers listed years in their current position. PD providers self-rated EBP knowledge using a 1 to 5 Likert scale (1 = strongly disagree to 5 = strongly agree) in response to the statement “I have the knowledge to increase use of EBPs for autism within my organization.” The “high EBP knowledge” variable was created by dichotomizing EBP knowledge responses, such that “strongly agree” responses were coded as “1” and all other responses were coded as “0”. This variable was dichotomized to capture high EBP expertise for the PD providers. They also rated EBP authority using a 1 to 5 Likert scale (1 = strongly disagree to 5 = strongly agree) in response to the statement “I have the authority to increase the use of EBPs for autism within my organization.” The “high EBP authority” variable was created by dichotomizing EBP authority responses, such that “strongly agree” responses were coded as “1” and all other responses were coded as “0”. This variable was dichotomized to capture high authority to insure high level influence over financial matters at the district level.

### 2.6. Data Analysis

Four General Linear Models (GLM) were conducted to explore the relationship between PD provider performance and identified predictors. The outcome variable for PD provider performance was a continuous variable. All four models included three PD providers characteristics, including a continuous variable for the number of years in professional role, a dichotomous variable for high EBP knowledge and a dichotomous variable for high EBP authority and two key SELPA characteristics, including multiple districts (with single district as the reference group) and dummy variables for medium and large district size (with small district size as the reference group). Model 2 added the network structure predictor, network size and the density of the high communication network. Network size was constrained to fewer than 8 people in order to reduce subject burden. This constraint likely had minimal impact due to a network size of 8 being greater than 3 standard deviations above average. Model 3 included the number of EBP experts and Model 4 included coaching network and size of financial support network. All variables were assessed for normality and any violations of the distributional assumptions.

## 3. Results

Comparisons of means revealed no statistically significant variation in network variables, however, there were differences in means across contexts. Significance tests were constrained by network size, which was capped at eight and by the overall small network size. The PD providers’ overall network size varied by context, with the largest overall network size in large districts (mean 1.94) and the smallest in medium-sized districts (mean 1.56). The density of closeness among alters was similar across contexts. The number of alters with EBP expertise was highest in large districts (mean 1.10) and lowest in medium-sized contexts (mean 0.81). The number of people with financial authority in the network was highest in small districts and lowest in medium-sized districts. The number of people with coaching supporters in the network was highest in large districts and lowest in medium-sized districts. See Table 2.

Four multiple linear regression models were fit to predict performance scores. For PD providers, high EBP knowledge was associated with performance across all models. Two social network variables were associated with PD performance, including a higher number of alters rated as having high levels of EBP expertise and larger coaching networks. Social network variables were tested in four separate models, in order to avoid issues of multicollinearity (ρ > 0.8). See Table 3.

## 4. Discussion

Understanding the complexities of the research-to-practice gap requires investigation of multiple factors related to the system (Aarons et al. 2011). While our analyses do not include evaluations from teachers, families or students about PD provider performance due to financial constraints for the study, we are able to identify influential factors from the self-reports of PD providers. At the PD provider level, EBP knowledge was key to increased implementation of high-quality coaching and training practices, while years in their position and EBP authority were not. This is useful as a potential mechanism since it is much easier to influence knowledge than experience or authority. Implications in practice contradict current practices used for identifying and selecting PD providers. District leaders often hire PD providers because of their years of experience. Our findings suggest that EBP knowledge is an essential characteristic of effective PD providers, more so than years of experience.

The size of the coaching networks influenced performance outcomes over and above greater system-level characteristics, indicating the importance of networks for PD providers. While this is the first analysis testing the impact of the networks of higher-level PD providers on their performance as coaches for classroom teachers of autistic students, this aligns with the existing literature on the impact that networks have on teacher, principal and district leader networks (Coburn 2001; Coburn and Russell 2008; Daly and Finnigan 2012, 2013; Penuel et al. 2009; Rodway 2019; Spillane 2005). Additionally, it appears that networked coaching support is more important than networked financial support for PD providers performance. Although these data were collected from within one state public education system, the implications for PD providers can be applied more broadly. For example, publicly provided educational services within the US and internationally often rely on regionalized expertise for program development and improvements. Educational professionals in PD provider roles will likely benefit from support networks, regardless of location and organizational structures (Rodway 2019). Future research should explore this complex issue.

Future studies should look at how professional networks might be used as an intervention to improve the performance of PD providers. This study suggests that increased personal knowledge about EBPs, increased knowledge of network members who are also knowledgeable about EBPs and larger coaching networks all appear to have some positive influence on the performance of PD providers. These factors could be integrated into an intervention specifically for PD providers. For example, an effective intervention for PD providers might include the establishment of a community of practice where PD providers are intentionally connected with others who are highly knowledgeable in their content area and are able to share advice and support related to their training and coaching efforts within their educational agencies. This is one possible implication for these findings; however, future research may identify additional practical applications for increasing professional networks and tailoring interventions for this targeted outcome.

## 5. Limitations

One potential limitation of the current study is that all of the data are self-reported. Identifying and establishing objective metrics to look at factors related to EBP knowledge, self-efficacy, quantity and quality of training and coaching, fiscal contributions to training and coaching efforts, as well as frequency of contact with people in each type of network could further develop our understanding of how networks influence professional development and the use and scale of EBPs for autism. Due to the high correlations between the network metrics, it is difficult to determine what component of the network (number of EBP, coaching network, overall size) has the most impact on the relationship between the networks and the performance scores.

## 6. Conclusions

Our findings indicate that PD providers who had larger networks overall, larger coaching support networks and more network members with EBP expertise had higher levels of performance in their work as trainers and coaches. This study suggests that social network size and quality at a systems level can influence the quality of education for autistic children who require evidenced based interventions. Building larger support networks for PD providers coaching teachers in classrooms may have potential as an intervention to improve EBP use and scale up in educational settings.

## Figures and Tables

**Table 1.** Variable descriptive statistics.

	Mean (SD)/Number (%)	
Performance Outcome		
Provider Performance score	41.89	(19.01)
PD Provider Characteristics		
Years in Position	6.25	(5.25)
High EBP Knowledge	0.58	(58%)
High EBP Authority	0.35	(35%)
Context Characteristics		
Multi-District SELPA^1^	151	(67%)
Single-District SELPA	75	(33%)
Large SELPA	108	(48%)
Medium SELPA	69	(31%)
Small SELPA	49	(21%)
Support Network Predictor		
*Network Structure*		
Overall Network Size	1.79	(1.65)
High Communication Density	0.22	(0.38)
*Network Resources*		
# EBP^2^ Experts	1.21	(1.32)
# of Coaching providers	1.10	(1.35)
# of Finance providers	1.00	(1.07)
# CAPTAIN^3^ PD^4^ providers/Leaders	0.78	(1.11)

1 SELPA = Special Education Local Plan Area.

2 EBP = Evidence-based practices for Autism Spectrum Disorder.

3 CAPTAIN = California Autism Professional Training and Information Network.

4 PD = Professional Development.

**Table 2.** PD provider network characteristics.

	PD Provider Social Network Variables				
	Multi-District (*N* = 151)	Single District (*N* = 75)	Large (*N* = 108)	Medium (*N* = 69)	Small (*N* = 49)
*Social Network Variables*	Mean	Mean	Mean	Mean	Mean
***Network Types***					
Network Structure					
Overall Network Size	1.85	1.66	1.94	1.56	1.79
Communication Density	0.42	0.45	0.47	0.36	0.45
Network Resources					
# EBP^1^ Experts in Network	1.04	0.89	1.10	0.81	1.02
# Coaching Supporters in Network	1.23	1.17	1.32	1.10	1.24
# Financial Authority in Network	1.14	1.01	1.14	0.95	1.20
# CAPTAIN^2^ in Network	0.79	0.76	0.94	0.57	0.73
PD^3^ providers Performance	41.89	41.87	42.62	41.88	40.27

1 EBP = Evidence-based practices for Autism Spectrum Disorder.

2 CAPTAIN = California Autism Professional Training and Information Network.

3 PD = Professional Development.

**Table 3.** CAPTAIN PD providers performance success models.

	Outcome: Professional Development Performance Score							
	(1)		(2)		(3)		(4)	
	Mean	sd	Mean	sd	Mean	sd	Mean	sd
Intercept	32.665 ***	3.508	28.893 ***	3.613	28.540 ***	3.554	28.644 ***	3.597
PD^1^ Provider Predictors								
Years in Position	0.154	0.235	0.185	0.23	0.191	0.228	0.202	0.229
High EBP^2^ Knowledge	12.784 ***	2.640	12.272 ***	2.586	12.200 ***	2.560	12.069 ***	2.574
High EBP Authority	0.018	2.753	−1.069	2.712	−0.492	2.669	−1.010	2.694
SELPA^3^ Characteristics								
Multi-Districts	−0.545	2.674	−0.861	2.617	−0.594	2.589	−1.060	2.604
Large SELPA	1.880	3.223	1.695	3.152	1.627	3.121	2.139	3.136
Medium SELPA	0.826	3.401	1.482	3.331	1.695	3.300	1.899	3.320
Network Structure								
Network Size			2.428 ***	0.731				
Network High Density			−1.827	8.173				
Size * Density			0.757	3.188				
Network Resources								
# EBP People					3.545 ***	0.898		
# of Financial							1.138	1.203
# of Coaches							2.954 ***	0.951
Observations	226		226		226		226	
R^2^	0.112		0.155		0.171		0.168	
Adjusted R^2^	0.087		0.120		0.144		0.137	
Residual Std. Error	18.161 (df = 219)		17.838 (df = 216)		17.585 (df = 218)		17.658 (df = 217)	
F Statistic	4.595 *** (df = 6; 219)		4.397 *** (df = 9; 216)		6.425 *** (df = 7; 218)		5.475 *** (df = 8; 217)	

1 PD = Professional Development.

2 EBP = Evidence-based practices for Autism Spectrum Disorder.

3 SELPA = Special Education Local Plan Area.

* < 0.1;

*** < 0.01.

## Data Availability

Data is not publicly available.
